# Body Mass Index Influences the Prognostic Impact of Combined Nuclear Insulin Receptor and Estrogen Receptor Expression in Primary Breast Cancer

**DOI:** 10.3389/fendo.2017.00332

**Published:** 2017-11-28

**Authors:** Sofie Björner, Ann H. Rosendahl, Maria Simonsson, Andrea Markkula, Karin Jirström, Signe Borgquist, Carsten Rose, Christian Ingvar, Helena Jernström

**Affiliations:** ^1^Faculty of Medicine, Department of Clinical Sciences Lund, Oncology and Pathology, Lund University, Lund, Sweden; ^2^Clinical Trial Unit, Forum South, Skåne University Hospital, Lund, Sweden; ^3^CREATE Health and Department of Immunotechnology, Lund University, Lund, Sweden; ^4^Department of Clinical Sciences Lund, Surgery, Lund University, Skåne University Hospital, Lund, Sweden

**Keywords:** nuclear insulin receptor, estrogen receptor alpha, body mass index, breast cancer, prognosis, adjuvant breast cancer treatment

## Abstract

The prognostic importance of tumor-specific nuclear insulin receptor (InsR) expression in breast cancer is unclear, while membrane and cytoplasmic localization of InsR is better characterized. The insulin signaling network is influenced by obesity and may interact with the estrogen receptor α (ERα) signaling. The purpose was to investigate the interplay between nuclear InsR, ER, body mass index (BMI), and prognosis. Tumor-specific expression of nuclear InsR was evaluated by immunohistochemistry in tissue microarrays from 900 patients with primary invasive breast cancer without preoperative treatment, included in a population-based cohort in Sweden (2002–2012) in relation to prognosis. Patients were followed for up to 11 years during which 107 recurrences were observed. Nuclear InsR^+^ expression was present in 214 patients (23.8%) and increased with longer time between surgery and staining (*P* < 0.001). There were significant effect modifications by ER status and BMI in relation to clinical outcomes. Nuclear InsR^+^ conferred higher recurrence-risk in patients with ER^+^ tumors, but lower risk in patients with ER^−^ tumors (*P*_interaction_ = 0.003). Normal-weight patients with nuclear InsR^+^ tumors had higher recurrence-risk, while overweight or obese patients had half the recurrence-risk compared to patients with nuclear InsR^−^ tumors (*P*_interaction_ = 0.007). Normal-weight patients with a nuclear InsR^−^/ER^+^ tumor had the lowest risk for recurrence compared to all other nuclear InsR/ER combinations [HR_adj_ 0.50, 95% confidence interval (CI): 0.25–0.97], while overweight or obese patients with nuclear InsR^−^/ER^−^ tumors had the worst prognosis (HR_adj_ 7.75, 95% CI: 2.04–29.48). Nuclear InsR was more prognostic than ER among chemotherapy-treated patients. In summary, nuclear InsR may have prognostic impact among normal-weight patients with ER^+^ tumors and in overweight or obese patients with ER^−^ tumors. Normal-weight patients with nuclear InsR^−^/ER^+^ tumors may benefit from less treatment than normal-weight patients with other nuclear InsR/ER combinations. Overweight or obese patients with nuclear InsR^−^/ER^−^ tumors may benefit from more tailored treatment or weight management.

## Introduction

Overweight and obesity confer poor breast cancer prognosis such as increased risk for recurrence and death among patients with high body mass index (BMI) compared to lower BMI (1–4). There is an established connection between obesity and the insulin signaling pathway (5). One animal study indicated that the level of nuclear translocation of insulin receptor (InsR) may differ according to body weight (6). Whether the relationship between overweight/obesity and poor breast cancer prognosis is partly mediated through nuclear localization of InsR is unknown. The InsR is a tyrosine kinase receptor and belongs to the same receptor subfamily as the insulin-like growth factor 1 receptor (IGF1R). The two receptors share structural homology, signal *via* similar downstream signaling pathways, can form heterodimers, and both receptors are implicated in breast cancer (7, 8). The cytoplasmic and membrane tumor expression of InsR alone and in combination with IGF1R and phospho-IGF1R/InsR in relation to breast cancer outcome has previously been described in the same patient cohort as used in the present study. Patients with tumors expressing high abundance of IGF1R and InsR and concomitant activation were associated with the worst prognosis (9). In addition, a significant association between longer time between surgery and staining and weaker cytoplasmic and membrane InsR and phospho-IGF1R/InsR staining was reported.

Most studies of InsR have focused on the cytoplasmic and membranous localization of InsR, and mRNA levels of InsR including the two splice variants InsR-A and InsR-B and their associations with breast cancer features and prognosis (10, 11). It is well known that InsR internalization and downstream signaling activation of PI3K and AKT is followed by either receptor recycling to the membrane or lysosomal degradation (12, 13). In addition, InsR can translocate to the nucleus. Although not fully understood, both InsR and IGF1R have genomic functions involving the mitogen-activated protein kinase signaling pathway and cellular functions such as proliferation and migration (14–17). The IGF1R and estrogen receptor (ER) signaling pathways are subject to feedback cross-talk and are closely linked (18, 19). In a cell study, nuclear InsR-expression was higher in ERα-depleted breast cancer cells and was able to suppress the IGF1R promotor activity *in vitro* (16, 20). Others have shown that the expression of hormone receptors such as ER was linked to BMI (21, 22) and that higher BMI was linked to higher estradiol levels in breast tissue in patients with ER^+^ but not ER^−^ tumors (23). Estrogen synthesis occurs though aromatization of androgens to estrogens by CYP19A1 in the adipose tissue, both locally in the breast as well as peripherally (3). In women, ligand levels are influenced by BMI. A higher BMI was associated with higher levels of circulating IGF-1, proinsulin, insulin, and C-peptide (24, 25). Studies in adipocytes and hepatocytes indicated that the internalization of the InsR only occurred after ligand stimulation (26, 27). Further, obesity was associated with hyperinsulinemia and caused abnormalities in the insulin signaling pathway (5), which may impact the expression and localization of insulin-related factors, such as InsR, in breast cancer. Host factors such as BMI are therefore of importance in studies of prognostic and treatment predictive markers in breast cancer. Several treatment strategies directed against the IGF-1 axis are in clinical trials, but have mostly been disappointing in the clinical setting (28). Better prognostic and treatment predictive biomarkers are warranted. Neither the association between nuclear localization of InsR and patient’s BMI or tumor ER status, nor the prognostic importance of the nuclear InsR have been established among breast cancer patients. In this study, we hypothesized that the prognostic value of tumor-specific nuclear InsR expression may differ according to the patients’ BMI, tumor ER status, as well as type of adjuvant treatment.

## Materials and Methods

### Patients

The breast cancer specimens were obtained from primary breast cancer patients in an ongoing population-based cohort, BC Blood Study, consisting of 1,116 patients. The patients were included between October 2002 and June 2012 at the Skåne University Hospital, Lund, Sweden as previously described (29). The included patients were between 24 and 99 years old and had no previous history of cancer within the last ten years. A research nurse measured the patients’ anthropometric factors: weight, height, waist and hip circumference, and breast volume (30, 31) prior to surgery. In addition, patients were asked to fill in questionnaires regarding lifestyle factors as well as reproductive history and use of exogenous hormones or other medications during the past week. Patients were classified as diabetic if they self-reported use of any kind of antidiabetic treatment.

Clinicopathological data and information about breast cancer recurrences and deaths were obtained from medical records, pathology reports, the Regional Tumor Registry and the Population Registry (32–34). Adjuvant breast cancer treatment was administered according to standard of care and was recorded until the first breast cancer event. In patients without any breast cancer events, treatments were recorded until last follow-up or death prior to July 1, 2014. Patients may have received more than one type of adjuvant treatment during follow-up. As of November 2005, human epidermal growth factor receptor 2 (HER2) evaluation was introduced and trastuzumab was included in the adjuvant treatment setting. These were entered as a covariate in subgroup analyses of patients. Aromatase inhibitors (AIs) are mainly offered to postmenopausal patients and the impact of nuclear InsR on AI-treatment response was therefore only analyzed among patients ≥50 years.

This study was carried out in accordance with the recommendations of the local ethics committee at Lund University with written informed consent from all patients. All patients gave written informed consent in accordance with the Declaration of Helsinki. The protocol was approved by the local ethics committee at Lund University (Dnr 75-02, Dnr 37-08, Dnr 658-09, Dnr 58-12, Dnr 379-12, Dnr 227-13, Dnr 277-15, and Dnr 458-15).

The final study cohort consisted of 900 patients after excluding patients who received preoperative treatment (*n* = 51), patients with only ductal carcinoma *in situ* (*n* = 39), patients with distant metastasis ≤0.3 years from baseline (*n* = 8), or patients with no evaluable invasive tumor tissue on tissue microarray (TMA) (*n* = 118). The report followed the Reporting recommendations for tumor MARKer prognostic studies (REMARK) criteria (35).

### TMA and Immunohistochemistry

Representative tumor regions of formalin-fixed paraffin-embedded tissue blocks were collected from surgical specimens and assembled in a TMA containing duplicate 1.0 mm cores, using a semi-automated tissue array device (Beeches instruments, Sun Prairie, WI, USA). Freshly cut sections were automatically deparaffinized and pretreated using the PT Link system (DAKO, Glostrup, Denmark). All slides were stained simultaneously in one batch to obtain identical conditions in order to minimize staining variation due to technical factors. Immunohistochemistry was performed using the Autostainer Plus from DAKO with the EnVision FLEX high-pH kit, according to the manufacturer’s instructions (DAKO, Glostrup, Denmark) with the InsR (β-subunit) (GR36, Calbiochem; dilution 1:50) antibody. The InsR antibody detects the β subunit, which is identical for the isoforms InsR-A and InsR-B. InsR expression in the present study thus reflects the total amount of InsR. ERα was routinely stained for clinical purposes at the Department of pathology in Lund and ERα status was obtained from pathology reports (32–34). In Sweden, an ERα cutoff of >10% of stained nuclei is still used in the clinic compared to the 1% cutoff that is used in other countries.

The immunohistochemical staining was evaluated by two independent observers (SBj, AR) blinded to tumor characteristics and patient information. Reexamination was performed in case of discrepancy (1.5%) until consensus was reached. The evaluated tumors were divided into negative or any positive nuclear staining. In case of bilateral tumors, all scores were based on the same tumor when evaluating the combined nuclear, cytoplasmic and membrane InsR expression. The prognostic impact of the cytoplasmic and membrane InsR staining intensities have been reported previously (9).

### Statistical Methods

All statistical analyses were calculated using he SPSS software versions 22 (IBM). χ^2^ tests were used for analyses between dichotomized expression levels in different cellular compartments. Logistic regression tests were used for analyses between expression levels and categorical variables, presenting both crude odds ratios (OR) and adjusted OR (OR_adj_) for time between surgery and staining (TBSAS, years). Kaplan–Meier curves and LogRank tests were used for univariable survival analyses. Cox regression was used for multivariable analyses providing hazard ratios (HRs) with 95% confidence intervals (CIs) adjusted for age (continuous), invasive tumor size (≥21 mm or skin or muscular involvement), any axillary lymph node involvement, histological grade III, ER status, BMI (≥25 kg/m^2^), TBSAS (years), and treatments. Two-way interaction terms between nuclear InsR and BMI (≥25 kg/m^2^), and between nuclear InsR and ER status, and three-way interaction terms between nuclear InsR, BMI (≥25 kg/m^2^), and ER status were calculated and used in adjusted Cox regression analyses to investigate potential effect modifications. In case of bilateral tumors (*n* = 15), the highest score was applied. The tumor characteristics from the same tumor were used in all analyses where tumor characteristics were included. Sensitivity analyses were performed using the scores for the contralateral invasive tumor. Restriction analyses were performed where patients who reported pre-operative treatment with anti-diabetic medications were excluded.

Breast cancer events were defined as local or regional recurrences, contralateral cancer or distant metastasis, and the time to event is referred to as event-free survival (EFS). For distant metastasis-free survival (DMFS), only distant metastases were considered events, and death due to any cause was the only considered event for overall survival (OS). Patients without events were censored at the last follow-up or death prior to July 1, 2014. Patients were followed from inclusion to the first breast cancer event, distant metastasis, or death, respectively.

Power calculations assuming 900 patients with an accrual interval of 10 years and additional follow-up time of two years and a frequency of 25% of positive nuclear InsR tumors showed that the study was able to detect true HRs between 0.762 and 1.342 with 80% power and an α of 5% (36). All *P*-values presented are two-tailed and nominal and were not adjusted for multiple testing since this is an exploratory study. A *P*-value of less than 0.05 was considered significant.

## Results

### Correlations between Localization of InsR in Different Cellular Compartments and Year of Surgery

**Figure 1:**
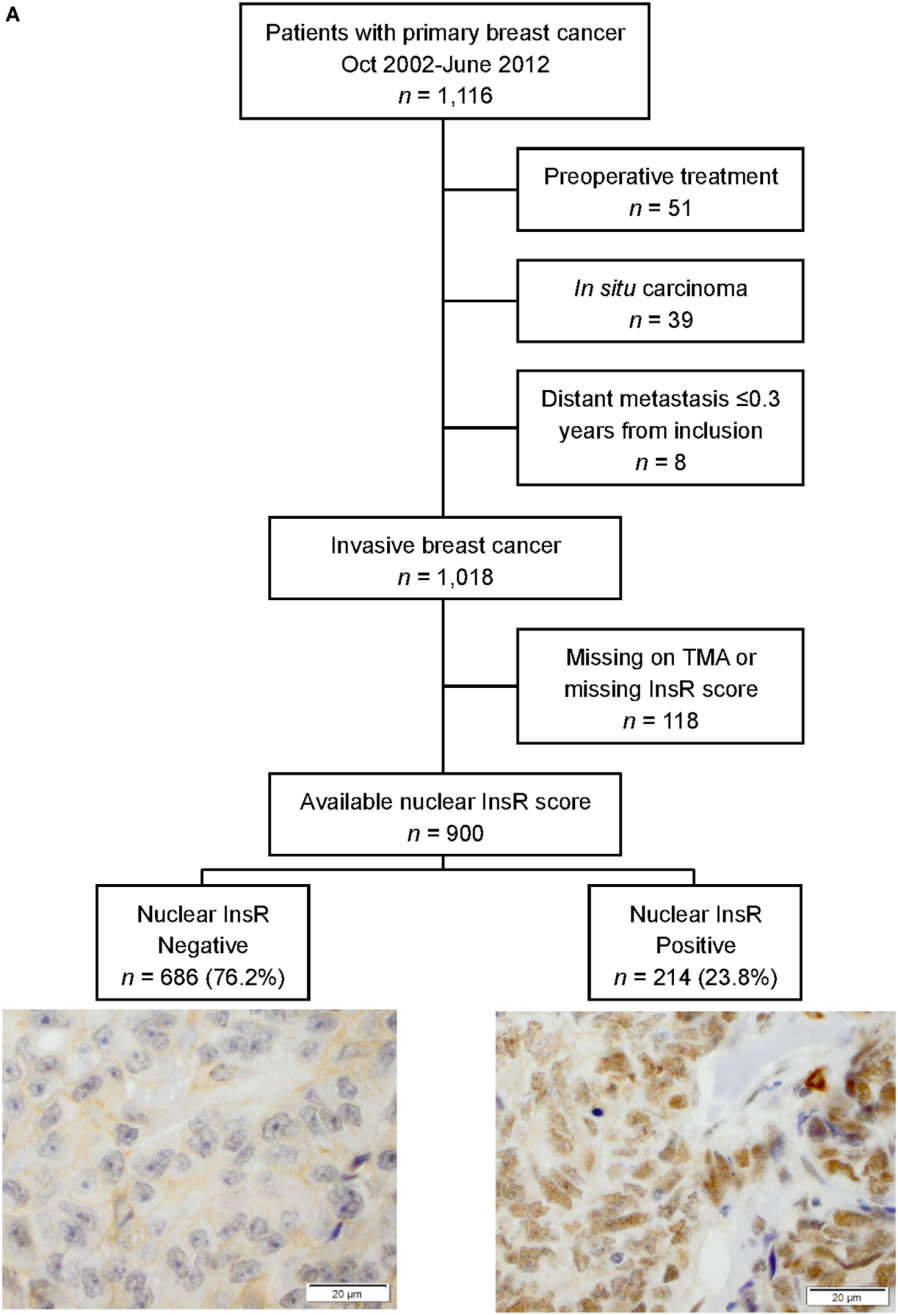

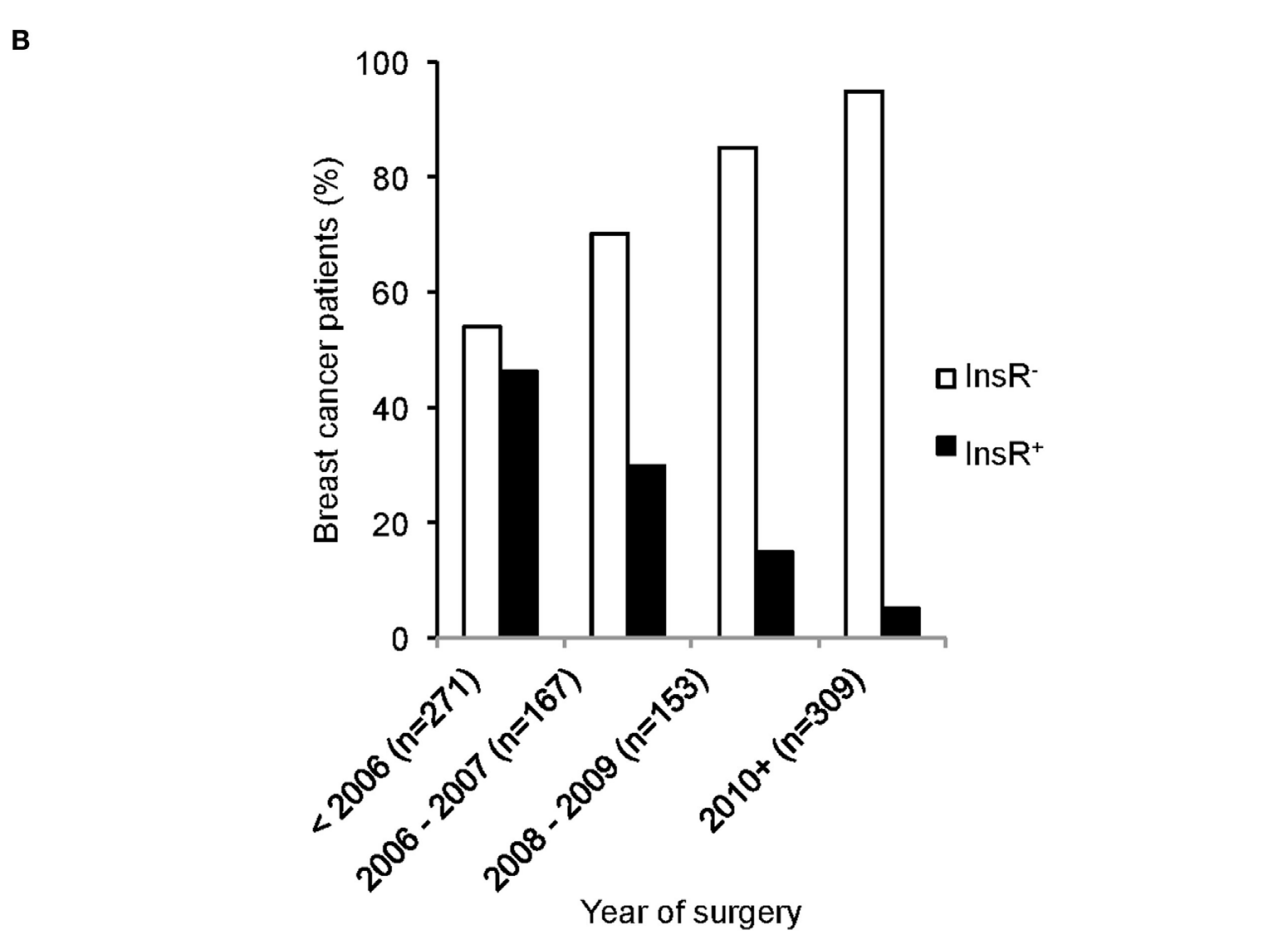
**(A)** Flow chart of study population and marker distribution with representative images of negative and positive nuclear insulin receptor (InsR) expressions, scale bar = 20 µm. **(B)** Nuclear InsR expression in relation to year of surgery.

Figure 1A illustrates the distribution of nuclear InsR staining. There were 900 tumors (88.4%) with available nuclear InsR scores of which 214 (23.8%) tumors were positive for nuclear InsR. There was a significant association between longer TBSAS and positive nuclear staining: nuclear InsR^+^: 9.0 years (IQR 7.0–10.0, *P* < 0.001), nuclear InsR^−^: 5.0 years (IQR 3.0–8.0), indicating that the proportion of patients with nuclear InsR^+^ tumors decreased over time with the highest proportion observed among patients included before 2006 (Figure 1B). There were significant inverse correlations in all time intervals between nuclear expression of InsR and membrane expression (negative, positive; all *r*_s_ ≤ −0.15, all *P-*values ≤ 0.047). For cytoplasmic intensity (negative, weak, moderate, strong) and dichotomized cytoplasmic InsR expression (negative/weak versus moderate/strong) there were inverse correlations that were significant in all time intervals with the exception of the years 2008–2009 (all *r*_s_ ≤ −0.12, all *P*-values ≤ 0.048). InsR was rarely present in all three compartments simultaneously.

### Nuclear InsR in Relation to Tumor and Patient Characteristics

Tables 1 and 2 present both crude and TBSAS adjusted associations between patient and tumor characteristics and nuclear InsR expression. In the crude analyses in Table 1, nuclear InsR^+^ was significantly associated with lower weight, BMI, and waist-to-hip ratio, as well as younger age. These factors were no longer significant after adjustment for TBSAS. In both the crude and the adjusted models in Table 2, nuclear InsR^+^ was significantly positively associated with HER2 amplification and postoperative adjuvant trastuzumab treatment, but not with ER^+^ status. Nuclear InsR^+^ was also significantly positively associated with postoperative adjuvant chemotherapy in the adjusted model.

**Table 1:** Patient characteristics in relation to tumor-specific expression of nuclear InsR.

			Patients with available tumor nuclear InsR status				Missing nuclear InsR status
	All, *n* = 1,018, median (IQR) or %	Missing total, *n*	Nuclear InsR^–^, *n* = 686 (76.2%), median (IQR) or %	Nuclear InsR^+^, *n* = 214 (23.8%), median (IQR) or %	Crude *P*-value^a^ or OR^a^ (95% CI) for InsR^+^	Adjusted *P*-value^b^ or OR^b^ (95% CI) for InsR^+^	*n* = 118, median (IQR) or %
**Patient characteristics**							
Age at diagnosis (years)	61.1 (52.1–68.1)	0	61.8 (52.7–69.0)	59.6 (50.7–65.5)	**0.010**	0.11	60.7 (49.6–68.1)
Weight (kg)	69.0 (62.0–78.0)	26	70.0 (62.0–80.0)	68.0 (61.5–76.0)	**0.050**	0.48	69.0 (62.0–77.0)
Height (m)	1.65 (1.62–1.70)	26	1.65 (1.62–1.70)	1.66 (1.62–1.70)	0.80	0.26	1.66 (1.62–1.69)
BMI (kg/m^2^)	25.1 (22.5–28.3)	28	25.3 (22.5–28.7)	24.7 (22.4–27.7)	**0.037**	0.25	24.8 (22.5–28.0)
Waist-to-hip ratio	0.86 (0.81–0.90)	38	0.86 (0.81–0.91)	0.84 (0.80–0.89)	**0.017**	0.95	0.86 (0.80–0.90)
Total breast volume, ≥850 mL (%)	57.3	160	55.6	60.2	1.21 (0.86–1.70)	1.16 (0.80–1.66)	62.5
Age at menarche (years)	13.0 (12.0–14.0)	6	13.0 (12.0–14.0)	13.0 (12.0–14.0)	0.86	0.96	13.5 (13.0–14.0)
Parous (%)	88.1	1	88.2	88.3	1.01 (0.63–1.63)	1.29 (0.78–2.16)	87.3
Age at first full-term pregnancy (years)	25.0 (22.0–28.0)	128	25.0 (22.0–28.0)	25.0 (21.0–28.0)	0.93	0.83	25.0 (22.0–27.0)
Ever use of oral contraceptives (%)	71.0	1	70.4	70.6	1.00 (0.72–1.41)	1.13 (0.79–1.63)	75.4
Ever treatment for menopausal symptoms (%)	44.0	3	44.3	45.5	1.05 (0.77–1.43)	0.97 (0.69–1.35)	39.8
Current smoker prior to surgery (%)	20.3	2	20.0	18.7	0.92 (0.62–1.36)	0.82 (0.54–1.24)	24.6
Alcohol abstainer (%)	10.5	7	10.6	10.7	1.02 (0.62–1.67)	0.94 (0.55–1.61)	9.3
Coffee consumption ≥ 2 cups/day (%)	81.3	4	80.5	81.3	1.05 (0.71–1.56)	0.94 (0.61–1.43)	85.6

*^a^Logistic regression, crude*.

*^b^Logistic regression adjusted for time between surgery and staining (TBSAS, years)*.

*IQR, interquartile range*.

*Bold font indicates statistically significant differences*.

**Table 2:** Tumor characteristics and treatments in relation to tumor-specific expression of nuclear InsR.

			Patients with available tumor nuclear InsR status				Missing nuclear InsR status

	All, *N* = 1,018, *n* (%)	Missing total, *n*	Nuclear InsR^–^, *n* = 686 (76.2%), *n* (%)	Nuclear InsR^**+**^, *n* = 214 (23.8%), *n* (%)	Crude OR^a^ (95% CI) for InsR^**+**^	Adjusted OR^b^ (95% CI) for InsR^**+**^	*n* = 118, *n*
**Tumor characteristics**							
Invasive tumor size		0					
≤20 mm	741 (72.8)		498 (72.6)	154 (72.0)	Ref.	Ref.	89
≥21 mm and skin or muscular involvement independent of size	277 (27.2)		188 (27.4)	60 (28.0)	1.03 (0.73–1.45)	1.17 (0.81–1.70)	29
Axillary lymph node involvement		2					
0	627 (61.7)		426 (62.3)	121 (56.5)	Ref.	Ref.	80
1–3	303 (29.8)		201 (29.4)	72 (33.6)	1.27 (0.93–1.73)	1.30 (0.93–1.82)	30
≥4	86 (8.5)		57 (8.3)	21 (9.8)			8
Histologic grade		1					
I	255 (25.1)		153 (22.3)	65 (30.4)	Ref.	Ref.	37
II	505 (49.7)		351 (51.2)	100 (46.7)			54
III	257 (25.3)		182 (26.5)	49 (22.9)	0.82 (0.57–1.18)	1.11 (0.75–1.64)	26
Histologic type		64					
Ductal	766 (80.3)		518 (80.4)	170 (85.4)	Ref.	Ref.	78
Lobular	111 (11.6)		77 (12.0)	15 (7.5)	0.59 (0.33–1.06)	0.56 (0.30–1.05)	19^c^
Other/mixed	77 (8.1)		49 (7.6)	14 (7.0)	0.87 (0.47–1.62)	0.66 (0.34–1.30)	14^c^
Hormone receptor status							
ER^+^ (>10%)	894 (87.9)	1	609 (88.9)	182 (85.0)	0.71 (0.46–1.11)	0.69 (0.42–1.12)	103
PR^+^ (>10%)	722 (71.0)	1	492 (71.8)	146 (68.2)	0.84 (0.60–1.17)	0.91 (0.63–1.30)	84
HER2 amplification^d^	83 (12.2)	49	46 (9.0)	18 (19.6)	**2.45 (1.35–4.46)**	**2.82 (1.48–5.37)**	19^c^
**Treatment by last follow-up^e^**							
Ever chemotherapy	259 (25.4)	0	177 (25.8)	51 (23.8)	0.90 (0.63–1.29)	**1.51 (1.01**–**2.25)**	31
Ever radiotherapy	641 (63.0)	0	439 (64.0)	135 (63.1)	0.96 (0.70–1.32)	1.02 (0.72–1.43)	67
Ever trastuzumab^d^	65 (8.9)	0	37 (6.8)	14 (14.3)	**2.30 (1.19–4.44)**	**3.39 (1.66–6.91)**	14^c^
ER^+^ only							
Ever endocrine therapy	695 (77.7)	0	480 (78.8)	143 (78.6)	0.99 (0.66–1.48)	0.95 (0.61–1.47)	72
Ever tamoxifen	528 (59.1)	0	357 (58.6)	115 (63.2)	1.21 (0.86–1.71)	0.81 (0.55–1.19)	56
Ever aromatase inhibitor	346 (38.7)	0	230 (37.8)	78 (42.9)	1.24 (0.88–1.73)	1.25 (0.86–1.80)	38

*^a^Logistic regression, crude*.

*^b^Logistic regression adjusted for time between surgery and staining (TBSAS, years)*.

*^c^Significant difference between included and excluded patients*.

*^d^HER2 was routinely analyzed in patients <70 years with invasive tumors as of November 2005 (*n* = 732)*.

*^e^Patients may have received more than one type of treatment prior to any event*.

*Bold font indicates statistically significant differences*.

### Nuclear InsR Expression in Relation to BMI and Year of Surgery

There was an inverse correlation between nuclear InsR expression and TBSAS in all three patients groups stratified by BMI status as normal-weight, overweight, and obese (<25.00, 25.00–25.99, 30.00+; all *r*_s_ ≤ −0.31, all *P*-values < 0.001). In contrast, there were no associations between nuclear InsR and BMI in the different time intervals (<2006, 2006–2007, 2008–2009, 2010+), indicating that the association between nuclear InsR and BMI did not change over time.

### Nuclear InsR As a Prognostic Marker Alone and in Relation to BMI

Patients were followed for up to 11 years with a median follow-up of 5.0 years for patients still at risk. Of the 900 patients, 107 had had any breast cancer event and 67 of these patients had a distant metastasis. Eighty-six patients died due to any cause and 52 of these patients had had a reported breast cancer event prior to death.

Nuclear InsR was not a prognostic marker for EFS, DMFS, or OS among all patients. However, there were significant effect modifications depending on BMI on the associations between nuclear InsR and EFS (*P*_interaction_ = 0.007), DMFS (*P*_interaction_ = 0.040), and OS (*P*_interaction_ = 0.027). After adjustment for prognostic factors and TBSAS, normal-weight patients with nuclear InsR^+^ tumors had a non-significant increased risk for any breast cancer event and non-significant shorter DMFS and OS. In contrast, overweight or obese patients with nuclear InsR^+^ tumors had a significantly decreased risk of any breast cancer event [adjusted HR (HR_adj_) 0.48, 95% CI: 0.25–0.92], and of death due to any cause (HR_adj_ 0.35, 95% CI: 0.17–0.72), and a non-significant longer DMFS.

### Combined Nuclear InsR/ER As Prognostic Marker

There were also significant effect modifications of ER status on the association between nuclear InsR and EFS where nuclear InsR^+^ expression in ER^+^ tumors was associated with a non-significant higher risk of any breast cancer event and with lower risk in patients with ER^−^ tumors (*P*_interaction_ = 0.003). A similar effect modification between nuclear InsR and ER status was observed regarding DMFS (*P*_interaction_ = 0.043), but not with respect to OS. A combined nuclear InsR/ER expression score for each tumor was therefore calculated resulting in four different groups: InsR^−^/ER^+^ (*n* = 609), InsR^+^/ER^+^ (*n* = 182), InsR^+^/ER^−^ (*n* = 32), and InsR^−^/ER^−^ (*n* = 76). One patient had missing ER status.

#### Prognostic Impact of Nuclear InsR/ER Expression

**Figure 2:**
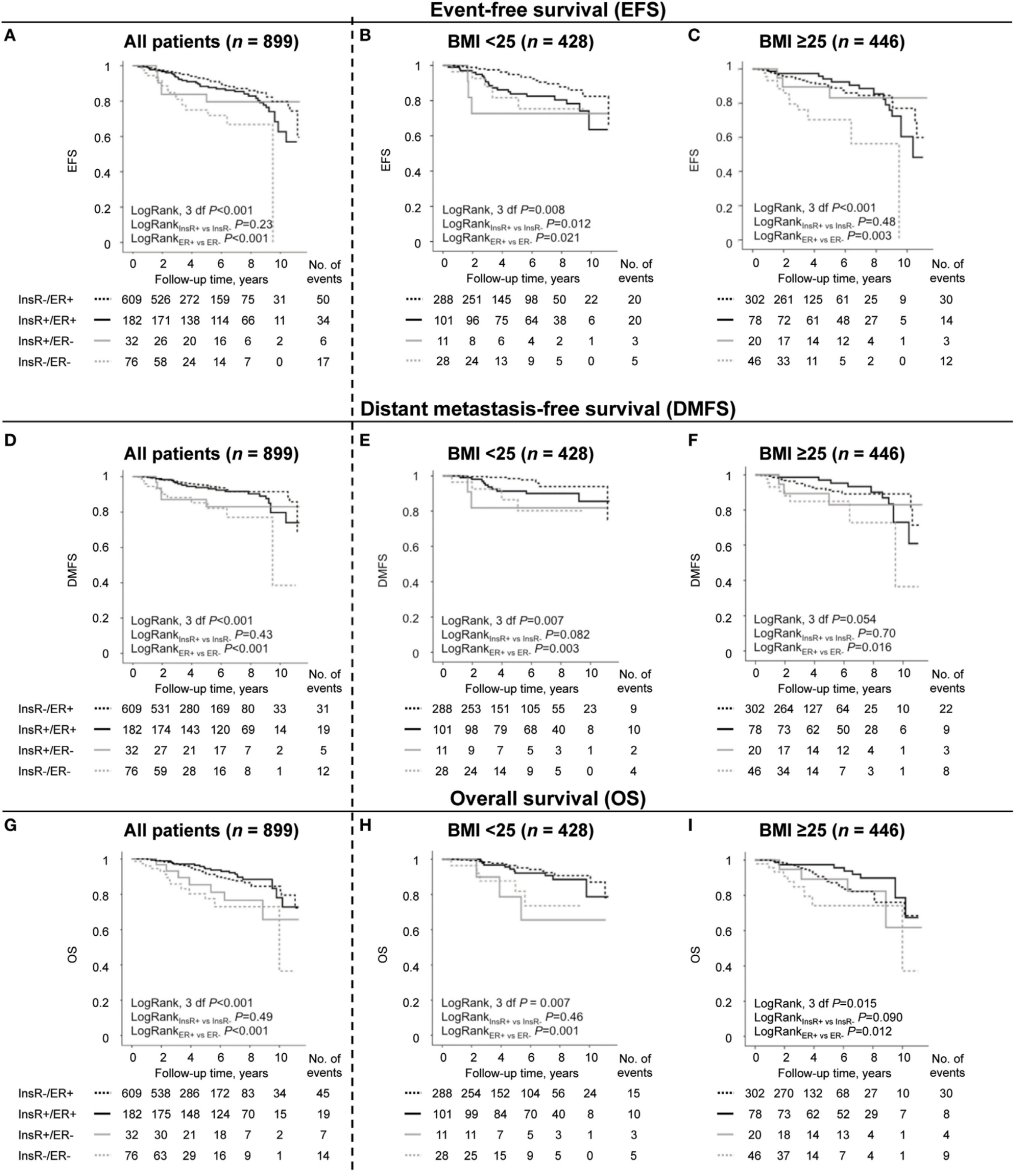
The prognostic importance of expression of nuclear insulin receptor (InsR)/estrogen receptor (ER) combinations among all patients (*n* = 899) and in normal-weight patients with body mass index (BMI) < 25 kg/m^2^ (*n* = 428) and in overweight or obese patients with BMI ≥ 25 kg/m^2^ (*n* = 446) regarding **(A–C)** event-free survival (EFS), **(D–F)** distant metastasis-free survival (DMFS), and **(G–I)** overall survival (OS), (*n*_missing_ = 25 for BMI and *n*_missing_ = 1 for ER status).

The independent prognostic impact of nuclear InsR alone and in combination with ER and BMI on EFS is presented in different multivariable models in Supplementary Table 1. Overall, among all patients before and after BMI stratification, ER status seemed to impact prognosis more than nuclear InsR status (Figure 2), except among normal-weight patients regarding EFS (Figure 2B). However, irrespective of BMI, patients with nuclear InsR^−^/ER^−^ tumors had, compared to all other nuclear InsR/ER combinations, the worst EFS (HR_adj_ 5.03, 95% CI: 1.74–14.54; Figure 2A) and DMFS (HR_adj_ 3.57, 95% CI: 1.04–12.23; Figure 2D), but not OS (Figure 2G).

#### Prognostic Impact of Nuclear InsR/ER in Relation to BMI

After stratification by BMI, the increased risk was mainly limited to the overweight or obese group of patients with nuclear InsR^−^/ER^−^ tumors compared to all other nuclear InsR/ER combinations (HR_adj_ 7.75, 95% CI: 2.04–29.48; Figure 2C). Overweight or obese patients with a nuclear InsR^−^/ER^−^ tumors had also highest risk of DMFS (HR_adj_ 3.90, 95% CI: 0.97–15.74; Figure 2F) and OS (HR_adj_ 2.72, 95% CI: 0.79–9.35; Figure 2I) compared to all other nuclear InsR/ER combinations. Conversely, overweight or obese patients with nuclear InsR^+^/ER^+^ tumors had a significantly lower risk for death (HR_adj_ 0.39, 95% CI: 0.17–0.89) compared to all other nuclear InsR/ER combinations. Normal-weight patients with a nuclear InsR^−^/ER^+^ tumor, which constituted two thirds of the patients, had the lowest risk for recurrence compared to all other nuclear InsR/ER combinations (HR_adj_ 0.50, 95% CI: 0.25–0.97; Figure 2B). There was no significant three-way interaction between nuclear InsR, ER, and BMI with respect to EFS when a formal three-way interaction analysis was performed.

### The Prognostic Impact of Combined Nuclear InsR/ER or Individual Nuclear InsR in Relation to Adjuvant Treatment and BMI Status

The combined expression of nuclear InsR and ER was investigated in relation to prognosis in different breast cancer treatment groups. For endocrine treatment, only patients with ER^+^ tumors were included. Any breast cancer event was used as a marker for poor adjuvant treatment response.

#### Chemotherapy

**Figure 3:**
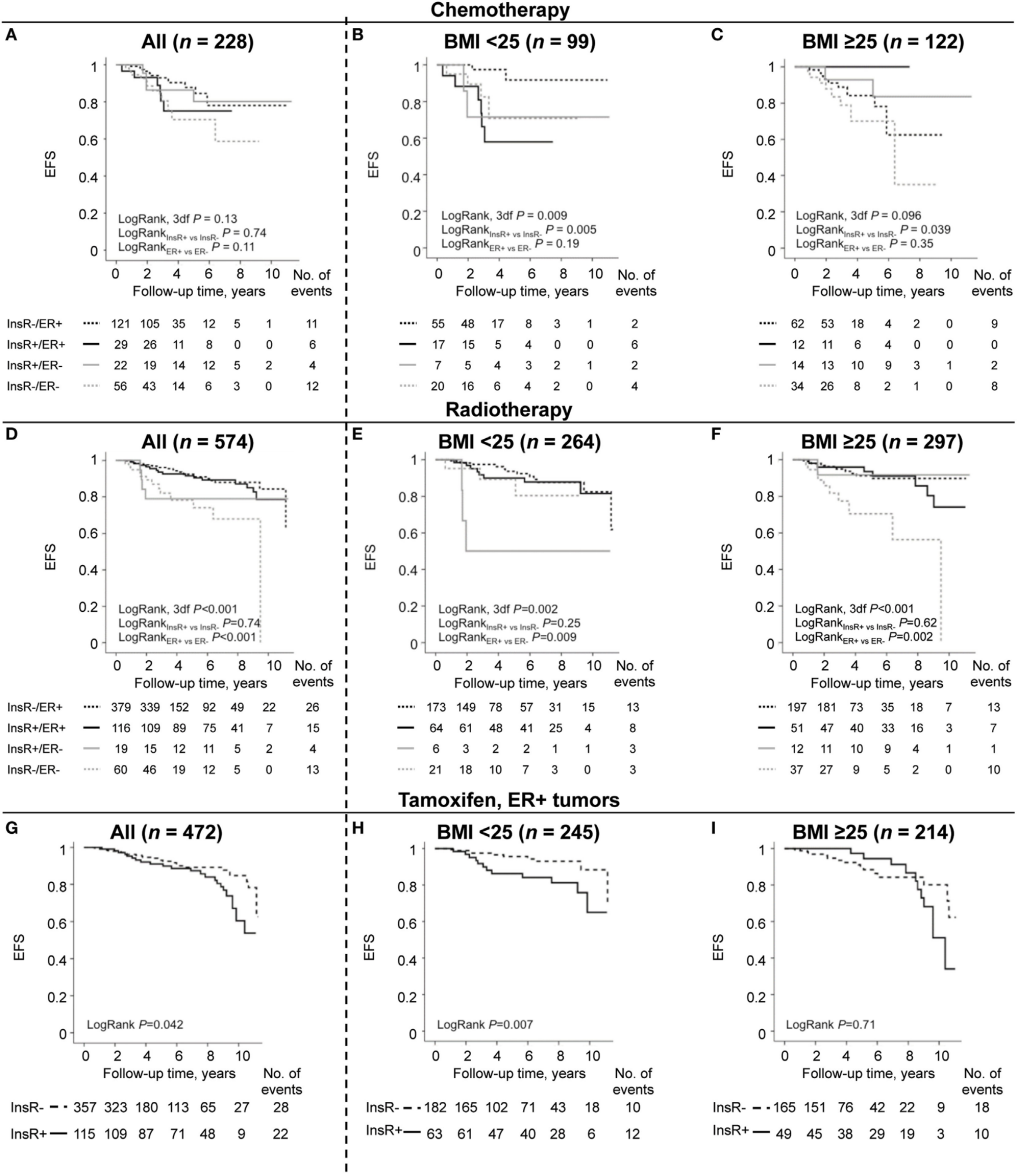
Combinations of nuclear insulin receptor (InsR)/estrogen receptor (ER) as prognostic marker for event-free survival (EFS) for all patients and in patients stratified by body mass index (BMI). **(A–C)** Chemotherapy-treated patients, **(D–F)** radiotherapy-treated patients, and **(G–I)** tamoxifen (TAM)-treated patients with ER^+^ tumors.

There were significant effect modifications of BMI on the association between nuclear InsR and EFS (*P*_interaction_ = 0.003), and DMFS (*P*_interaction_ = 0.047), but not for OS. Overweight or obese chemotherapy-treated patients with nuclear InsR^+^-expressing tumors had a significantly lower risk of any event (HR_adj_ 0.12, 95% CI: 0.02–0.69), while opposite results were observed among chemotherapy-treated normal-weight patients with nuclear InsR^+^-expressing tumors (HR_adj_ 2.61, 95% CI: 0.76–9.00). In all chemotherapy-treated patients, combined nuclear InsR/ER was not a prognostic marker for EFS (Figure 3A), DMFS, or OS. The lowest risk for recurrence was again observed among normal-weight patients with nuclear InsR^−^/ER^+^ tumors compared to all other nuclear InsR/ER combinations (HR_adj_ 0.08, 95% CI: 0.01–0.52; Figure 3B), while overweight or obese patients with nuclear InsR^−^/ER^−^ tumors had the worst prognosis compared to all other nuclear InsR/ER combinations (HR_adj_ 6.66, 95% CI: 1.14–38.94; Figure 3C). In conclusion, nuclear InsR conferred more prognostic information than ER status in chemotherapy-treated patients after stratification by BMI. Overweight or obese patients with nuclear InsR^−^/ER^−^ tumors had the worst prognosis compared to other groups.

#### Radiotherapy

In radiotherapy-treated patients overall, combined nuclear InsR/ER was a prognostic marker for EFS (Figure 3D), DMFS and OS, but this was driven by ER status for all three outcomes. There was a borderline interaction between BMI and nuclear InsR for EFS (*P*_interaction_ = 0.10), and significant interactions for both DMFS (*P*_interaction_ = 0.038) and OS (*P*_interaction_ = 0.042). In normal-weight patients, three out of six patients with nuclear InsR^+^/ER^−^ tumors had an early event (Figure 3E), although not significant in the multivariable model. Similar to the overweight or obese chemotherapy-treated group, overweight or obese radiotherapy-treated patients with nuclear InsR^−^/ER^−^ tumors had the highest risk for recurrence compared to all other nuclear InsR/ER combinations (HR_adj_ 12.62, 95% CI: 1.51–105.27; Figure 3F). In summary, the combined prognostic impact of nuclear InsR/ER in radiotherapy-treated patients was predominantly due to the ER status. Overweight or obese patients with nuclear InsR^−^/ER^−^ tumors again had the worst prognosis compared to other groups.

#### Endocrine Treatment

Among tamoxifen (TAM)-treated patients with ER^+^ tumors, nuclear InsR^+^ expression was weakly associated with shorter EFS (Figure 3G), although not significant in the multivariable model. There was a borderline significant effect modification depending on BMI between nuclear InsR and EFS (*P*_interaction_ = 0.10). Multivariable survival analyses revealed that nuclear InsR only had prognostic impact among normal-weight TAM-treated patients. A significant increased risk of any breast cancer event was seen among normal-weight patients with nuclear InsR^+^ tumors (HR_adj_ 2.84, 95% CI: 1.10–7.32; Figure 3H) but not among overweight or obese patients (Figure 3I). In all TAM-treated patients with ER^+^ tumors, nuclear InsR^+^ tumors were associated with a 2-fold increased risk for shorter DMFS (HR_adj_ 2.30, 95% CI: 1.02–5.19). However, as observed for EFS, this increased risk was limited to normal-weight patients (HR_adj_ 3.70, 95% CI: 1.01–13.60), while there was no increased risk in overweight or obese patients (*P*_interaction_ = 0.12). No significant associations were observed between nuclear InsR and OS in TAM-treated patients.

There was a borderline significant interaction between BMI and nuclear InsR on EFS (*P*_interaction_ = 0.069) but not on DMFS or OS among AI-treated patients aged ≥50 years. The effect estimates were similar to those seen in TAM-treated patients. However, nuclear InsR expression was not an independent prognostic marker for EFS, DMFS, or OS among AI-treated patients aged ≥50 years, irrespective of BMI status.

In brief, in normal-weight TAM-treated patients with ER^+^ tumors, nuclear InsR may confer poor prognosis, but not in patients with higher BMI.

### Sensitivity and Restriction Analyses

Sensitivity analyses were performed due to the 15 patients with available bilateral tumors. Nine patients had evaluable invasive tumor tissue on the contralateral side and five tumors changed dichotomized status for the patients with two evaluable tumors. The effect estimates remained essentially the same in all sensitivity analyses using the dichotomized status for the contralateral tumor. In total there were six analyses that went from significant to borderline significant and three analyses that went from borderline to significant.

There were 29/898 patients (3.2%) who self-reported any kind of antidiabetic treatment based on the preoperative questionnaire (two missing) who were excluded in the restriction analyses. Among these 29 patients, there were two breast cancer events, no distant metastasis, and nine deaths. The frequency of nuclear InsR was non-significantly lower in patients who reported anti-diabetic treatment (13.8%) compared with other patients (24.2%; *P* = 0.20). There were ten analyses that changed from significant to borderline significant and four analyses that changed from borderline to significant, but the effect estimates remained similar.

## Discussion

This study suggests that nuclear localization of InsR has differential prognostic roles depending on the patients’ BMI and ER status in contrast to membrane and cytoplasmic localized InsR (9). These findings are important since obesity is a global health concern associated with aberrant insulin sensitivity and also impaired breast cancer outcomes. InsR can translocate to the nucleus and act as a transcription factor, but the role of nuclear expressed InsR in breast cancer prognosis has to our knowledge not been previously investigated. In the current study, BMI only modified the association between nuclear InsR and clinical outcome, but did not impact the association between membrane and cytoplasmic InsR expression and clinical outcome. *In vitro* findings suggest that nuclear localization of InsR is higher in ER-depleted cells than in ER^+^ cells (16), but in the present study there was no association between tumor-specific nuclear InsR and ER status in contrast to membrane and cytoplasmic InsR expression (9). Further, only the nuclear InsR expression was associated with HER2 amplification in contrast to membrane and cytoplasmic InsR expression (9).

Insulin receptor is part of the IGF-signaling network and there is crosstalk between this network and other signaling pathways such as ER (20, 37, 38). The current study suggests that nuclear InsR may be of prognostic importance among normal-weight patients with ER^+^ tumors and among overweight or obese patients with ER^−^ tumors. Potential explanations for the observed differences in prognostic impact of nuclear InsR according to BMI include changes in methylations patterns (39), ligand levels (5, 23–25, 40), and altered intracellular receptor trafficking and recycling rates depending on ligand levels (41), as well as differential nuclear InsR suppression of the IGF1R promotor according to tumor ER status (16). These potential mechanisms may in part explain the observed effect modifications of BMI and ER on the clinical impact of nuclear InsR and need to be elucidated in future studies. Age and menopausal status also impact BMI (4), but in this study there was no significant interaction between nuclear InsR and age ≥50 years (data not shown). Patients were therefore not stratified based on age. Finally, in addition to ER status, breast cancer is a heterogeneous disease with multiple subtypes (42), which may modify the prognostic importance of nuclear InsR.

Insulin has the ability to enhance the cytotoxic effect of chemotherapy *in vitro* (43), which may explain the observed decreased recurrence-risk among overweight or obese chemotherapy-treated patients with nuclear InsR^+^ tumors in this study. The interplay between InsR, ER and IGF1R emphasizes the importance of combining the expression of nuclear InsR and ER and their impact as prognostic and treatment predictive biomarkers needs further elucidation. Targeting of all three receptors may be more efficient since dual inhibition of InsR and IGF1R with tyrosine kinase inhibitor OSI-906 in combination with ER downregulator fulvestrant more effectively suppress hormone-independent tumor growth than either drug alone (44). The current study indicates that BMI may also yield additional treatment predictive information with respect to drugs targeting the IGF1R/InsR and ER pathways. Since there is an ongoing obesity epidemic, nuclear InsR, which appears to play a differential role in overweight or obese compared to normal-weight patients, warrants further investigation in an independent study, preferably in randomized clinical trials of IGF-targeting treatments.

One of the findings in this study was that nuclear InsR expression was associated with time between surgery and staining. Changes of the expression of immunohistochemical markers over time should be considered in the statistical modeling when evaluating the prognostic impact of new markers. There was a significant association between earlier year of surgery and positive nuclear InsR staining. However, nuclear expression was inversely correlated with cytoplasmic and membrane InsR expression, indicating that the total InsR expression was stable over time, while the localization of InsR differed. The fact that the total InsR expression was stable over time may be considered as an internal control and validate the immunohistochemical staining. Additionally, all immunohistochemistry was performed simultaneously in one single batch in order to minimize technical variation. There may be several explanations for different InsR localizations such as differences in pre-analytical handling of the tumor tissue and differences in the standard of care for the patients, including different glucose concentrations in the perioperative intravenous drip or different anesthetic agents. Use of betamethasone to counteract postoperative nausea may impact blood glucose levels (45, 46), which could potentially impact on InsR nuclear translocation (26, 27), but lies outside the scope of this study. The differences in nuclear InsR expression in relation to year of surgery have been taken into account in all the regression analyses where TBSAS have been adjusted for. The results were driven by patients included 2002–2007. There were few patients with nuclear InsR^+^ tumors 2008–2012 and the prognostic importance of this marker in today’s setting needs to be elucidated. In addition to adjustments for TBSAS, restriction and sensitivity analyses were also performed. The results remained essentially the same after exclusion of patients treated with anti-diabetic medications and in sensitivity analyses for the patients with bilateral tumor tissue on the TMA.

This is a population-based cohort with high follow-up rates (29, 47, 48). The Swedish population have in general a lower BMI compared to the North American population (49), which may impact the generalizability of the results. The vast majority of the patients’ body measurements were taken preoperatively by a research nurse, which minimizes the risk for bias. HER2 status was only available as of November 2005, which is a weakness. The ERα cutoff of >10% is still used in the clinic in Sweden and is higher than in other countries. However, over 85% of the patients still had ER^+^ tumors.

In summary, the results from this study support that the prognostic value of nuclear InsR expression depends on cellular localization and that the prognostic value of nuclear InsR is dependent on ER and BMI status. Furthermore, this study highlights the importance of investigating possible changes in staining intensity and encourages that future evaluation of immunohistochemical markers should take the time between surgery and staining into consideration. Our results substantiated that nuclear InsR expression had a higher prognostic value than ER among chemotherapy-treated patients once BMI was taken into account. Conversely, ER was a better prognostic marker of radiotherapy response than nuclear InsR. The study further suggests that nuclear InsR may be of prognostic importance among normal-weight patients with ER^+^ tumors and in overweight or obese patients with ER^−^ tumors. The normal-weight patients with nuclear InsR^−^/ER^+^ tumors may thus benefit from less treatment than normal-weight patients with other nuclear InsR/ER combinations. Overweight or obese patients with nuclear InsR^−^/ER^−^ had the worst prognosis of all patients. Whether they may benefit from more tailored treatment, weight management, or better control of blood glucose levels remains to be elucidated.

## Ethics Statement

This study was carried out in accordance with the recommendations of the local ethics committee at Lund University with written informed consent from all patients. All patients gave written informed consent in accordance with the Declaration of Helsinki. The protocol was approved by the local ethics committee at Lund University (Dnr 75-02, Dnr 37-08, Dnr 658-09, Dnr 58-12, Dnr 379-12, Dnr 227-13, Dnr 277-15, and Dnr 458-15).

## Author Contributions

Conception and design: SBj, AR, CR, CI, and HJ. Development of methodology and analysis and interpretation of data: SBj, AR, and HJ. Acquisition of data: SBj, AR, MS, AM, CI, and HJ. Administrative, technical, or material support: SBj, AR, MS, AM, KJ, and HJ. Study supervision: CI and HJ. Writing, review, and/or revision of the manuscript: SBj, AR, MS, AM, KJ, SB, CR, CI, and HJ.

## Conflict of Interest Statement

SB has consultant fee honoraria from Novartis and Roche.

## Supplementary Material

Figure S1 Representative images of cytoplasmic, membrane and nuclear InsR staining in breast cancer tissue, scale bar = 20 µm.
