# Supplementary figures and images for: Body Mass Index Influences the Prognostic Impact of Combined Nuclear Insulin Receptor and Estrogen Receptor Expression in Primary Breast Cancer
Source: Front Endocrinol (Lausanne). 2017 Nov 28;8:332. doi: 10.3389/fendo.2017.00332 (PMC5712344; doi:10.3389/fendo.2017.00332)

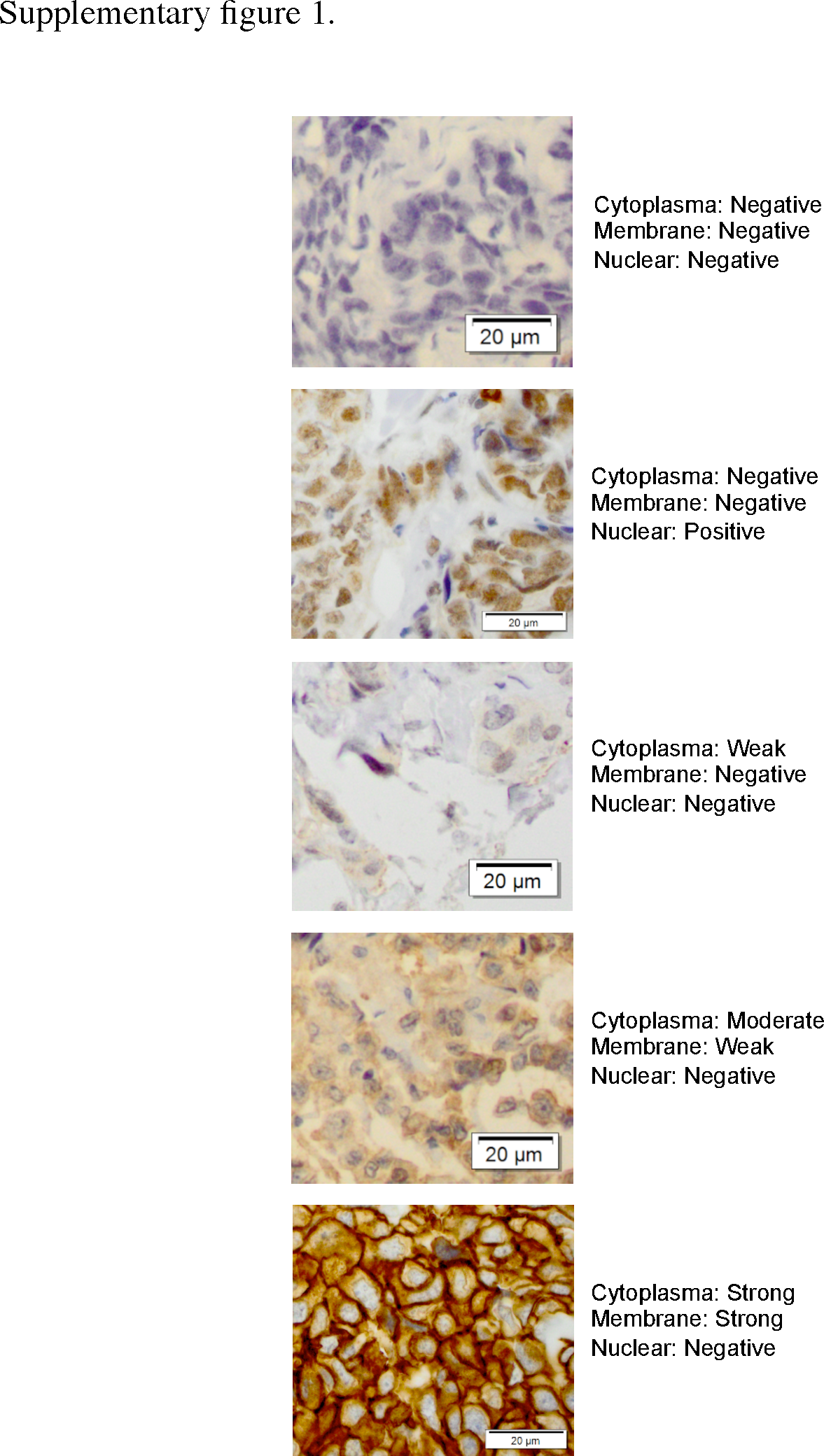

Supplement: Figure S1 — Representative images of cytoplasmic, membrane and nuclear InsR staining in breast cancer tissue, scale bar = 20 µm. [file image_1.tif]
