# Supplementary material for: Body Mass Index Influences the Prognostic Impact of Combined Nuclear Insulin Receptor and Estrogen Receptor Expression in Primary Breast Cancer
Source: Front Endocrinol (Lausanne). 2017 Nov 28;8:332. doi: 10.3389/fendo.2017.00332 (PMC5712344; doi:10.3389/fendo.2017.00332)
Supplement: Supplementary file 2 [file table_1.docx]

| **Supplementary table 1.** Multivariable Cox regression models for EFS by nuclear InsR alone and in combination with ER, for all patients and stratified by BMI. | | | | | | | | | | | | |
| --- | --- | --- | --- | --- | --- | --- | --- | --- | --- | --- | --- | --- |
|  |  |  |  |  |  | **Adjusted HR** | | | | | | |
|  |  |  |  | **Crude HR** |  | **Model 1** |  | **Model 2** |  | **Model 3** |  | **Model 4*** |
| **Tumor status** | **Total** | **Events** | **Missing** | **HR (95% CI)** |  | **HR_adj_^a^ (95% CI)** |  | **HR_adj_^a, b^ (95% CI)** |  | **HR_adj_^a, b, c^ (95% CI)** |  | **HR_adj_^a, b, c, d^ (95% CI)** |
|  | ***n*** | ***n*** | ***n*** |  |  |  |  |  |  |  |  | ***n*** |
| All | 900 |  |  |  |  |  |  |  |  |  |  | 646 |
| Nuclear InsR status |  |  | 0 |  |  |  |  |  |  |  |  |  |
| Nuclear InsR^-^ | 686 | 67 |  | Ref. |  | Ref. |  | Ref. |  | Ref. |  | Ref. |
| Nuclear InsR^+^ | 214 | 40 |  | 1.28 (0.86-1.90) |  | 1.10 (0.74-1.66) |  | 1.00 (0.66-1.51) |  | 0.93 (0.61-1.42) |  | 1.10 (0.53-2.30) |
| *BMI* |  |  | 25 |  |  |  |  |  |  |  |  |  |
| BMI<25 kg/m^2^ |  |  |  |  |  |  |  |  |  |  |  |  |
| Nuclear InsR^-^ | 316 | 25 |  | Ref. |  | Ref. |  | Ref. |  | Ref. |  | Ref. |
| Nuclear InsR^+^ | 112 | 23 |  | **2.06 (1.16-3.66)** |  | 1.78 (0.98-3.21) |  | 1.80 (0.98-3.29) |  | 1.71 (0.93-3.17) |  | 2.46 (0.75-8.15) |
| BMI ≥25 kg/m^2^ |  |  |  |  |  |  |  |  |  |  |  |  |
| Nuclear InsR^-^ | 349 | 42 |  | Ref. |  | Ref. |  | Ref. |  | Ref. |  | Ref. |
| Nuclear InsR^+^ | 98 | 17 |  | 0.81 (0.45-1.45) |  | 0.71 (0.39-1.27) |  | 0.59 (0.32-1.08) |  | **0.48 (0.26-0.92)** |  | 0.59 (0.19-1.82) |
| Combined nuclear InsR/ER |  |  | 1 |  |  |  |  |  |  |  |  |  |
| Nuclear InsR^-^/ER^+^ | 609 | 50 |  | Ref. |  | Ref. |  | Ref. |  | Ref. |  | Ref. |
| Nuclear InsR^+^/ER^+^ | 182 | 34 |  | 1.52 (0.98-2.37) |  | 1.32 (0.84-2.07) |  | 1.35 (0.85-2.12) |  | 1.30 (0.82-2.06) |  | 1.90 (0.83-4.36) |
| Nuclear InsR^+^/ER^-^ | 32 | 6 |  | 1.78 (0.76-4.15) |  | 1.58 (0.67-3.70) |  | 0.92 (0.37-2.29) |  | 0.48 (0.17-1.40) |  | 0.83 (0.16-4.24) |
| Nuclear InsR^-^/ER^-^ | 76 | 17 |  | **3.62 (2.08-6.31)** |  | **3.59 (2.06-6.26)** |  | **2.80 (1.50-5.22)** |  | 1.86 (0.88-3.97) |  | 2.58 (0.79-8.50) |
| *BMI* |  |  | 26 |  |  |  |  |  |  |  |  |  |
| BMI<25 kg/m^2^ | |  |  |  |  |  |  |  |  |  |  |  |
| Nuclear InsR^-^/ER^+^ | 288 | 20 |  | Ref. |  | Ref. |  | Ref. |  | Ref. |  | Ref. |
| Nuclear InsR^+^/ER^+^ | 101 | 20 |  | **2.25 (1.19-4.23)** |  | **1.95 (1.02-3.71)** |  | **2.03 (1.05-3.93)** |  | **2.02 (1.03-3.96)** |  | 2.62 (0.73-9.35) |
| Nuclear InsR^+^/ER^-^ | 11 | 3 |  | **4.11 (1.21-13.88)** |  | **3.49 (1.02-11.93)** |  | 2.37 (0.66-8.50) |  | 1.06 (0.24-4.64) |  | 6.10 (0.35-105.71) |
| Nuclear InsR^-^/ER^-^ | 28 | 5 |  | **2.99 (1.12-8.03)** |  | **2.99 (1.12-8.04)** |  | 2.46 (0.84-7.17) |  | 1.33 (0.39-4.49) |  | 3.52 (0.42-29.25) |
| BMI ≥25 kg/m^2^ | |  |  |  |  |  |  |  |  |  |  |  |
| Nuclear InsR^-^/ER^+^ | 302 | 30 |  | Ref. |  | Ref. |  | Ref. |  | Ref. |  | Ref. |
| Nuclear InsR^+^/ER^+^ | 78 | 14 |  | 1.01 (0.53-1.93) |  | 0.89 (0.46-1.70) |  | 0.87 (0.45-1.69) |  | 0.75 (0.38-1.49) |  | 1.05 (0.27-4.11) |
| Nuclear InsR^+^/ER^-^ | 20 | 3 |  | 0.99 (0.30-3.25) |  | 0.88 (0.27-2.91) |  | 0.57 (0.16-2.05) |  | 0.30 (0.07-1.41) |  | 0.51 (0.06-4.14) |
| Nuclear InsR^-^/ER^-^ | 46 | 12 |  | **3.76 (1.91-7.41)** |  | **3.69 (1.87-7.27)** |  | **3.11 (1.45-6.68)** |  | 2.45 (0.89-6.72) |  | 1.92 (0.40-9.20) |

NOTE: Events and missing data in the adjusted models: Model 1: 107 events in total, 48 and 59 events for BMI<25 and BMI ≥25, respectively; Model 2-3: 106 events, 47 events and 2 additional missing for BMI<25, and 59 events and 1 additional missing for BMI ≥25; Model 4: 43 events in total, 17 events for BMI<25, and 26 events and 1 additional missing for BMI ≥25.

* Patients included as of November 2005, *n*=646.

^a^ Adjusted for time between surgery and staining (TBSAS, years).

^b^ Adjusted for age (continuous), BMI (≥25 kg/m2), invasive tumor size (<21 mm vs. ≥21 mm or skin or muscular involvement independent of size), axillary lymph node involvement (yes/no), tumor grade III (yes/no), ER^+^ (yes/no).

^c^ Adjusted for treatment; chemotherapy, radiotherapy, TAM, and AIs.

^d^ Adjusted for trastuzumab treatment.
